# Hepatoprotective and Antioxidant Effects of Licorice Extract against CCl_4_-Induced Oxidative Damage in Rats

**DOI:** 10.3390/ijms12106529

**Published:** 2011-10-06

**Authors:** Hai Zhong Huo, Bing Wang, Yong Kang Liang, Yong Yang Bao, Yan Gu

**Affiliations:** Department of General Surgery, Ninth People’s Hospital affiliated to Shanghai Jiaotong University School of Medicine, Shanghai 200011, China; E-Mails: hzhhuodr7@sina.com (H.Z.H.); bwang5032@sina.com (B.W.); ykliangshyy1@sina.com (Y.K.L.); yybaodr28@sina.com (Y.Y.B.)

**Keywords:** licorice, CCl_4_, rat, antioxidant, AST, liver

## Abstract

Licorice has been used in Chinese folk medicine for the treatment of various disorders. Licorice has the biological capabilities of detoxication, antioxidation, and antiinfection. In this study, we evaluated the antihepatotoxic effect of licorice aqueous extract (LE) on the carbon tetrachloride (CCl_4_)-induced liver injury in a rat model. Hepatic damage, as reveled by histology and the increased activities of serum aspartate aminotransferase (AST), alanine aminotransferase (ALT), alkaline phosphatase (ALP) activities, and decreased levels of serum total protein (TP), albumin (Alb) and globulin (G) were induced in rats by an administration of CCl_4_ at 3 mL/kg b.w. (1:1 in groundnut oil). Licorice extract significantly inhibited the elevated AST, ALP and ALT activities and the decreased TP, Alb and G levels caused by CCl_4_ intoxication. It also enhanced liver super oxide dismutase (SOD), catalase (CAT), glutathione peroxidase (GSH-Px), glutathione reductase (GR), Glutathione S-transferase (GST) activities and glutathione (GSH) level, reduced malondialdehyde (MDA) level. Licorice extract still markedly reverses the increased liver hydroxyproline and serum TNF-α levels induced by CCl_4_ intoxication. The data of this study support a chemopreventive potential of licorice extract against liver oxidative injury.

## 1. Introduction

Liver is the key organ of metabolism and excretion. It is often exposed to a variety xenobiotics and therapeutic agents. Until today, people have not yet found an actual curative therapeutic agent for liver disorder. In fact, most of the available remedies help the healing or regeneration of the liver. The hepatotoxin carbon tetrachloride (CCl_4_) is frequently used to induce liver fibrosis in animal models [1]. Treatment with CCl_4_ generates free radicals that trigger a cascade of events that result in hepatic fibrosis, mimicking the oxidative stress that has a fibrogenic effect on HSC [2–4]. In fact, reactive oxygen species may cause tissue injury through activation of the precursors of MMPs (proMMPs) [2,4,5]. Although no successful therapeutic approach to this pathogenetic mechanism in liver disease has been developed, antioxidants therapies have shown to achieve some positive effects [6–8].

Natural remedies from medicinal plants are considered to be effective and safe alternative treatments for hepatotoxicity. In view of this, the present study was undertaken to investigate the hepatoprotective activity of licorice extract against CCl_4_ induced hepatotoxicity in male Wistar rats. Licorice has been used as a medicinal plant for thousands of years. The active component of licorice, glycyrrhizic acid, is hydrolyzed *in vivo* to glycyrrhetinic acid, which is responsible for most of its pharmacological properties. In ancient Chinese medicine and during Roman times, licorice was also recommended to cure sterility in women [9,10]. Licorice has held claim for therapeutic use for fevers, liver ailments, dyspepsia, gastric ulcers, sore throats, asthma, bronchitis, Addison’s disease and rheumatoid arthritis and has been used as a laxative, antitussive and expectorant [11–13]. Among its most consistent uses are as a demulcent for the digestive system, to treat coughs, to soothe sore throats, and as a flavoring agent.

The present study was undertaken to evaluate the protective effect of licorice aqueous extract on CCl_4_-induced hepatotoxicity. In addition, the antioxidant property of licorice aqueous extract in liver-injured rats was investigated.

## 2. Material and Method

### 2.1. Chemicals

Licorice was purchased from a local herb shop in Shanghai city, China in March 2011 and identified by Dr. X.J. Hu in College of Pharmacy, Shanghai Jiaotong University. A voucher specimen (No. 20110314) was deposited in the laboratory of this faculty. The plant material was dried at ambiant temperature and stored in a dry place prior to use.

Bifendate which is a recognised hepatoprotective drug in China was obtained from Zhejiang Wepon pharmacy Co. (Wenling, China). All other chemicals and reagents were of analytical grade.

### 2.2. Preparation of the Aqueous Extract of Licorice

The licorice root was washed well with water, dried at room temperature in the dark, and then ground in an electric grinder to give a coarse powder. A batch of 200 g of the licorice powder was suspended in 3 L of distilled water, and the mixture was boiled under reflux for 30 min. The decoction obtained was centrifuged at 8000 g for 10 min, and the supernatant was collected. The supernatant was then filtered with Whatman No. 1 filter paper to remove cellulose fibers. The filtrate was concentrated on a steam bath to give 60 g of the residue. The resulting residue was then dried using a lyophilizer (Four-Ring Science Instrument Plant, Beijing, Co., Ltd.). The yield of the lyophilized dry product was approximately 23% (w/w).

### 2.3. Animals

Wistar rats (200–250 g) of either sex were maintained under standard environmental conditions and had free access to feed and water. Experiments on animals were performed based on animal ethics guidelines of Institutional Animal Ethics Committee.

The rats were divided randomly into six groups of 10 rats. Four groups were administered with 100, 150, 300 mg/kg, of the licorice extract orally or bifendate (100 mg/kg) daily (positive control). The licorice extract was suspended in distilled water and administered orally through an intragastric tube at the dose of 100, 150, 300 mg/kg b.w. One group (*n* = 10) with the same number of rats served as a normal control and one group (*n* = 10) and the same number of rats served as a model control.

The selection of licorice extract dosage was based on our previous study wherein licorice extract administered at 300 mg/kg b.w./day for 15 days did not elicit any toxic symptoms in rats nor affected food intake or body weight gain. After 15 days, the rats (except for normal control) were given an acute oral dose of CCl_4_ at 3 mL/kg b.w. (1:1 in groundnut oil). The animals were housed individually in metallic cages at room temperature (25 ± 2 °C) with relative humidity of 50–60% and on a 12 h light–dark cycle. They had free access to food and water ad libitum. The food intake was monitored daily and the growth of animals was monitored weekly by recording body weight.

According to some Chinese medical literature [14], the dosage of licorice raw herb typically used in a clinic was about 10–30, or even 40 g in single use or in formulation. Excellent effect and no toxicity were observed in clinical practice. The result and dosage in the present study fit the clinical study, that is, if we use conversion table to compute the clinical dosage from the animal dosage, 100–300 mg/kg, it will be 10–40 g, near clinical usage. Hence, the licorice extracts at a dose of 100, 150 or 300 mg/kg BW were orally administered to rats injected with or without CCl_4_ treatment before CCl_4_ exposure.

The animals were sacrificed 8 h after CCl_4_ administration under light ether anesthesia. Blood was drawn by cardiac puncture for separation of serum. Liver organs were excised, blotted, weighed and processed for histopathology and biochemical assays. Ten or 20% (w/v) homogenates of the tissues were prepared in various buffers as per the requirement in specific assays.

Liver index was measured according to the formula: (rat liver weight/rat body weight) × 100%.

### 2.4. Histopathology

Liver tissues, which were previously trimmed into 2 mm thickness, were fixed with buffered formaldehyde for 24 h. The fixed tissues were processed including embedded in paraffin, sectioned and rehydrated. The histological examination by above conventional methods was evaluated the index of CCl_4_-induced necrosis by assessing the morphological changes in the liver sections stained with hematoxylin and eosin (H&E).

### 2.5. Biochemistry Analysis

ALT, AST, ALP activities, TP, Alb and G, TNF-α concentration were determined with Elisa Kits. Hydroxyproline has been used as an indicator to determine hepatic collagen amount. The tissue samples (50–60 mg) were hydrolyzed in 6 M HCl at 110 °C for 24 h. After filtration, samples were extracted and analyzed according to the procedure of Bergman and Loxley [15].

Reduced glutathione was determined according to Beutler’s method [16] using Ellman’s reagent. The procedure is based on the reduction of Ellman’s reagent by SH groups to form 5,5′-dithiobis (2-nitrobenzoic acid) with an intense yellow color, measured spectrophotometrically at 412 nm using a Shimadzu Spectrophotometer. Results were expressed as nmol GSH/mg protein.

Lipid peroxidation (LPO) levels in liver homogenates were estimated by Ledwozyw’s method [17]. In brief, the adduct formed, following boiled tissue homogenate with thiobarbituric acid, is extracted with *n*-butanol. The difference in optical density at 532 nm is measured in terms of the liver MDA content, also of thiobarbituric acid reactive substances (TBARS), which is undertaken as an index of lipid peroxidation. Results were expressed as nmol MDA/mg protein.

SOD was assayed by following the method of Misra and Frisovich [18]. Required amount (100 μL) of homogenate was added to tubes containing 0.5 mL of carbonate buffer and 0.5 of EDTA solution. The final volume was made up to 2.5 mL. The reaction was initiated by the addition of 0.5 mL of epinephrine and increase in absorbance at 480 nm was measured in a Systronics 119 UV spectrophotometer. One hundred percent auto oxidation of epinephrine to adrenochrome was performed in a control tube without the enzyme. The enzyme unit of activity was defined, as the enzyme required for 50% inhibition of epinephrine autooxidation.

CAT activity was measured by the method of Aebi [19]. A 0.1 mL of supernatant was added to cuvette containing 1.9 mL of 50 mM phosphate buffer (pH 7.0). Reaction was started by addition of 1.0 mL of freshly prepared 30 mM H_2_O_2_. The rate of decomposition of H_2_O_2_ was measured spectrophotometrically at 240 nm. Activity of catalase was expressed as U/mg of protein.

Activity of GSH-Px was determined according to the method of Lawrence and Burk [20]. The assay mixture consisted of 2.0 mL of 75 mM phosphate buffer (pH 7.0), 50 μL of 60 mM glutathione, 0.1 mL of 30 units/mL glutathione reductase, 0.1 mL of 15 mM EDTA, 0.1 mL of 3 mM NADPH and the appropriate amount of tissue supernatant to a final volume of 3.0 mL. The reaction was started by the addition of 0.1 mL of 7.5 mM H_2_O_2_. The rate of change of absorbance during the conversion of NADPH to NADP^+^ was recorded spectrophotometrically at 340 nm for 3 min. GSH-Px activity for tissues was expressed as μmoles of NADPH oxidized to NADP^+^ min^−1^ mg^−1^ protein.

GR was assayed using reagent from Randox Laboratories; the assay was adapted from the method of Goldberg and Spooner [21]. GR catalyzes the reduction of oxidized glutathione in the presence of NADPH, which is oxidized to NADP^+^. The decrease in absorbance is measured at 340 nm. One unit of activity is equal to the micromole of NADPH oxidized per minute per milligram of protein.

GST activity: Total GST activity was determined as described by Habig and Jakoby [22]. Briefly, the enzyme activity was assayed spectrophotometrically at 340 nm in a 4 mL cuvette containing 0.1 M PBS (pH 6.5), 30 mM glutathione, 30 mM 1-chloro-2,6-dinitrobenzene and tissue homogenate. Enzyme activity was expressed as nanomole per minute per milligram protein (nmol/min/mg protein).

### 2.6. Statistical Analysis

Statistical analysis involved used of the Statistical Analysis System software package. Analysis of variance was performed by ANOVA procedures. Significant differences between means were determined by Duncan’s multiple range tests at a level of *P* < 0.05.

## 3. Results

Toxicity test showed that 15 days of LE treatment did not significantly affect rats’ final body weight. There was no significant difference in average body weight gain and food utilization rate between groups (Table 1). None of experimental rats displayed toxic symptom and died. All rats had normal faeces.

**Figure 1:**
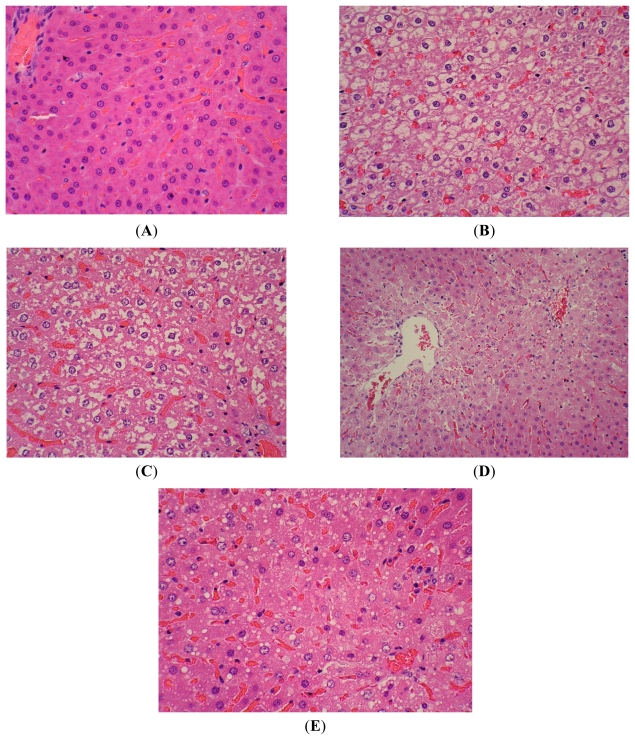
Histological Analysis of the Livers after CCl_4_ Administration. Typical images were chosen from the different experimental groups (original magnification 400×). (**A**) Control group; (**B**) vehicle-treated CCl_4_ group; (**C**) CCl_4_ and LE (150 mg/kg)-treated group; (**D**) CCl_4_ and LE (300 mg/kg)-treated group And (**E**) CCl_4_ and bifendate (100 mg/kg)-treated group.

Increased fatty degeneration, cytoplasmic vacuolization and necrosis provided histopathological evidence of tissue injury in the CCl_4_-treated group (Figure 1). However, these histopathologic indexes were significantly improved by LE and bifendate administration. CCl_4_ also increased the activities of serum ALT, ALT, ALP, and decreased the levels of serum TP, Alb and G in CCl_4_-treated rats compared to the normal control group (*p* < 0.01) (Table 2). Rats pre-treated with LE and bifendate showed a marked and significant reduction of serum ALT, ALT, ALP activities and increase of TP, Alb and G levels compared to the CCl_4_ group (*p* < 0.01) (Figure 1).

Table 3 showed livers indices in different groups of rats. In the CCl_4_ (model) group, the livers index was 1.5-fold higher than in the normal control group (*p* < 0.01). Treatment with the LE (100, 150, 300 mg/kg b.w.) reduced livers indices to 87%, 81% and 72%, respectively (*p* < 0.05, *p* < 0.01). In addition, treatment with the bifendate (100 mg/kg b.w.) reduced livers indices to 70% (*p* < 0.01).

Table 4 showed the liver hydroxyproline levels of rats in each group. There is a significant increase (*p* < 0.01) in the liver hydroxyproline levels of CCl_4_-induced rats (model group) when compared with the normal control group. Pre-treatment of LE (100, 150, 300 mg/kg b.w.) for 15 consecutive days dose-dependently significantly decrease (*p* < 0.01) the liver hydroxyproline levels in CCl_4_ + LE groups when compared with CCl_4_-treated group. In addition, pre-treatment with the bifendate (100 mg/kg b.w.) significantly reduced the liver hydroxyproline levels in bifendate-treated (CCl_4_ + bifendate) group when compared with CCl_4_-treated group.

To evaluate the protective effect of LE on the liver oxidative damage in rats induced by CCl_4_, we determined the primary antioxidant enzymes in liver. CCl_4_ significantly (*P* < 0.01) increased the concentrations of liver malondialdehyde (MDA) in CCl_4_-treated group (Table 5), which was product of lipid peroxidation, compared to that in normal control group. And the concentrations of liver MDA were dose-dependently reduced by the pre-administration of LE. As shown in Table 5, CCl_4_ markedly (*P* < 0.01) decreased the content of liver GSH which was reversed by LE in a dose-dependent manner. In addition, the content of GSH in the LE (100, 150, 300 mg/kg b.w.) treating (CCl_4_ + LE) groups was significantly (*P* < 0.05, *P* < 0.01) higher than that of normal control group.

As shown in Table 6, a significantly (*P* < 0.01) increased activities of liver antioxidant enzymes were observed in the CCl_4_-treated rats compared with the normal control group. Pre-feeding the LE at either 100, 150 or 300 mg/kg b.w. significantly (*P* < 0.05, *P* < 0.01) enhanced the liver antioxidant enzymes activities (SOD, CAT, GSH-Px, GR, GST) in licorice extract-treated (CCl_4_ + LE) rats in a dose-dependent manner. Likewise, pre-treatment of the bifendate (100 mg/kg b.w.) significantly (*P* < 0.01) elevated liver GSH level, SOD, CAT, GSH-Px, GST and GR activities, and decreased liver MDA level.

Table 7 showed serum TNF-α levels in different groups of rats. In the CCl_4_ (model) group, the serum TNF-α levels were 5-fold higher than in the normal control group (*p* < 0.01). Treatment with the LE (100, 150, 300 mg/kg b.w.) reduced serum TNF-α levels to 83%, 71% and 49%, respectively (*p* < 0.01). In addition, pre-treatment with the bifendate (100 mg/kg b.w.) reduced serum TNF-α levels to 50% (*p* < 0.01).

## 4. Discussion

Clinical benefits of licorice have been reported. Licorice extracts have been used in the clinical treatment of numerous illnesses (gastric and duodenal ulcers, bronchial asthma, infectious hepatitis, malaria, diabetes insipidus, contact dermatitis, *etc.*) with considerable success in China. The anti-inflammatory, mucoprotective and anti-ulcer activities of licorice make it an attractive treatment for peptic ulcer. Licorice may lower testosterone levels in women. Licorice root is hepatoprotective, antihepatotoxic, and has been used successfully in clinical trials to treat viral hepatitis [23,24].

The liver is a vital organ present in vertebrates and some other animals. It has a wide range of functions, including detoxification, protein synthesis, and production of biochemicals necessary for digestion [25,26]. A large number of plants and formulations have been claimed to have hepatoprotective activity. In China, many plants medicines possessed hepatoprotective activity. They included *Astragalus mongholicus*, *Salvia miltiorrhiza*, *Radix paeoniae alba*, *Angelica sinensis*, *Ligusticum wallichii*, *Ganodorma lucidum* and so on [14]. Compared with other herbs, because of wonderful pharmacological function, licorice was most widely, single use or in formulations, applied in therapy of many diseases, especially liver diseases in china. Previous studies showed that licorice extract could significantly reduce the elevated levels of LDH, GOT, GPT and MDA and increased the reduced levels of SOD and GSH-Px by CCl_4_ in carp [27]. Some formulation, like licorice *Radix paeoniae alba* decoction, *Glycyrrhiza*, *Salvia Chinensis Benth Zishen* decoction, could effectively protect rats against d-galactose-induced and CCl_4_-induced live injury.

Carbon tetrachloride (CCl_4_) has been widely used in animal models to investigate chemical toxin-induced liver damage. The most remarkable pathological characteristics of CCl_4_-induced hepatotoxicity are fatty liver, cirrhosis and necrosis, which have been thought to result from the formation of reactive intermediates such as trichloromethyl free radicals (CCl^3 +^) metabolized by the mixed function cytochrome p450 in the endoplasmic reticulum [28]. Usually, the extent of hepatic damage is assessed by the increased level of cytoplasmic enzymes (ALT, AST and ALP), thus leads to leakage of large quantities of enzymes into the blood circulation. This was associated by massive centrilobular necrosis, ballooning degeneration and cellular infiltration of the liver [29].

In this study, CCl_4_ treatments could modify liver function, since the activities of AST, ALP and ALT were significantly higher, and levels of TP, Alb and G were significantly lower than those of normal values as compared with the control group. In addition, liver index in the CCl_4_-treated rats were significantly higher than those of normal values as compared with the control group. This indicated that the model had been successfully built. It was more important to confirm whether there is any difference between the treatment with or without licorice extract under the damage of CCl_4_. The results showed that pre-treatment of rats with licorice extract effectively protected the animals against CCl_4_-induced hepatic destruction, as evidenced by decreased serum AST, ALT, and ALP activities and increased TP, Alb and G levels. In the histological examination, pre-treatment with licorice extract or bifendate suppressed the acute hepatic damage and was consistent with improvement of the serum biological parameters for hepatotoxicity.

CCl_4_ is a well-known hepatotoxic agent. The basis of its hepatotoxicity lies in its biotransformation by the cytochrome P450 system to two free radicals. The first metabolite, a trichloromethyl free radical, forms covalent adducts with lipids and proteins; it can interact with O_2_ to form a second metabolite, a trichloromethylperoxy free radical, or can remove hydrogen atoms to form chloroform [30]. Since free radicals play such an important role in CCl_4_-induced hepatotoxicity, it seems logical that compounds that neutralize such radicals may have an hepatoprotective effect. Indeed, various natural products have been reported to protect against CCl_4_-induced hepatotoxicity [31]. The medicinal herb Artemisia campestris has been found to scavenge free radicals and therein to exert a hepatoprotective effect against CCl_4_-induced liver injury [32]. Similarly, ginsenosides are reported to contribute to protection against CCl_4_-induced hepatotoxicity in rats, ostensibly by their antioxidant properties [33].

Antioxidant enzymes (SOD, GPx, GR, GST and catalase) represent one protection against oxidative tissue-damage [34]. SOD converted O_2_ into H_2_O_2_. GPx and catalase metabolize H_2_O_2_ to non-toxic products. The GSH antioxidant system plays a fundamental role in cellular defense against reactive free radicals and other oxidant species. This system consists of GSH and an array of functionally related enzymes, of which GR is responsible for the regeneration of GSH, whereas GPx and GST work together with GSH in the decomposition of hydrogen peroxide other organic hydroperoxides [35]. The results obtained indicate increased MDA levels in the liver in response to CCl_4_ treatment, implying increased oxidative damage to the liver. CCl_4_ also caused an increased in GPx, SOD, GR, GST and catalase activities in the liver over those of the control group. Under oxidative stress, some endogenous protective factors such as GPx and catalase are activated in the defense against oxidative injury [36]. For example, organophosphate pesticides such as dimethoate and malathion were shown to increase oxidative stress and consequently increase SOD and catalase activities in erythrocytes of rats [37]. This increase in enzyme activities was probably a response towards increased ROS generation in pesticides toxicity. In this work, licorice extract pre-treatments returned the increased MDA and decreased GSH, antioxidant enzymes levels back to their control levels, implying that licorice extract may prevent the peroxidation of lipids by CCl_4_.

Because antioxidants are reported to exert anti-inflammatory activity [38], we also evaluated the *in vivo* anti-inflammatory activity of licorice extract by measuring release of TNF-α, a pro-inflammatory cytokine that becomes elevated in acute and chronic diseases. Our work showed that pre-treatment of licorice extract could decrease serum TNF-α levels in CCl_4_-treated rats. TNF-α was found to mediate the induction of hepatotoxicity and fibrogenesis in the bile duct ligation model [39]. Phytochemicals have been shown to inhibit inflammation by blocking inflammatory pathways downstream of cytokine release [40], and also by reducing macrophage production of proinflammatory factors [41].

Bernardi *et al.* [42] evaluated the effects of graded doses of pure licorice root extract in order to identify the dosages leading to mineralocorticoid-like side effects. Two subjects from the 814 mg/day group were forced to withdraw from the study due to headaches, hypertension, hypokalemia and/or edema. Sigurjonsdottir *et al.* [43] monitored the effects of moderate consumption of licorice on blood pressure, plasma and urinary electrolytes, and urinary cortisol. He concluded that moderate licorice consumption can induce hypertensive effects, but the extent of these effects varies greatly between individuals. Moreover, licorice in combination with other components (*Coptis chinensis*, *Amur corktree bark*, *Corydalis tuber*, *Ephedra*, *etc*.) in prescription can promote effective components of medicines to precipitate, and decrease their biological availability in body. Heavy licorice (glycyrrhizin) consumption has been associated with shorter gestation [44]. Heavy glycyrrhizin exposure was associated with preterm delivery and may be a novel marker of this condition. Therefore, high dose of licorice should be cautiously used in clinic.

The main components of licorice root are the triterpene, saponins, glycyrrhizin/glyccyrrhizic acid and glycyrrhetic acid. Glycyrrhizic acid (GA) exhibits a number of pharmacological effects including anti-inflammatory and is used in hepatoprotective formulations. Pre-treatment with GA has been reported to show protective action against carbon tetrachloride (CCl_4_)-induced liver injury in rats [45]. Tsuruoka *et al.* [46] reported that glycyrrhizin (10.5 mg/kg) suppressed the increases in AST and ALT, inhibited iNOS mRNA expression and protein, cell infiltration and the degeneration of hepatocytes in the liver of concanavalin A (Con A)-treated mice. Lee *et al.* reported that glycyrrhizin alleviates CCl_4_-induced liver injury by diminishing free radical toxic properties and inducing heme oxygenase-1 and downregulating proinflammatory mediators [47]. Lin *et al.* reported that a three-day pretreatment with either glycyrrhizin or glycyrrhetinic acid exhibited protective effect on retrorsine-induced liver damage in rats [48]. Wan *et al.* reported that compared with glycyrrhizin or matrine alone, combined use of glycyrrhizin and matrine can reduce the mortality of acetaminophen overdosed mice, attenuate the development of acetaminophen-induced hepatotoxicity in mice, and reduce the number and area of γ-GT positive foci, thus protecting liver function and preventing hepatocellular carcinoma from occurring [49]. In addition, it has a protective effect on immunosuppression and a strong non-specific anti-inflammatory effect and an effect of reducing the incidence of sodium and water retention, without causing significant adverse effects. Saponins, triterpenes, sterols and bitter principles were identified in the *n*-hexane extract of *Lygodium flexuosum*. The *n*-hexane extract of *Lygodium flexuosum* was reported as a hepatoprotective agent against carbon tetrachloride-induced hepatotoxicity [50]. This indicated that saponins, triterpenes might possess hepatoprotective activities. We supposed that the components (triterpene, saponins, glycyrrhizic acid) in single or in combination with other components present in the licorice extract might be responsible for the reduction of hepatotoxicity in treatment groups.

Based on the experimental results reported here, we hypothesize that licorice extract may play an important role in medicine by scavenging free radicals, stimulating activities of antioxidant enzymes, and arresting production of inflammatory cytokine, subsequently protecting the liver against CCl_4_-induced damage. We supposed that the components (triterpene, saponins, glyccyrrhizic acid) in single or in combination with other components present in the licorice extract might be responsible for the reduction of hepatotoxicity in treatment groups.

## Figures and Tables

**Table 1:** Effect of licorice aqueous extract (LE) treatment on experimental rats’ average body weight gain and food utilization rate (*n* = 10).

Group	Initial body weight (g)	Final body weight (g)	Average body weight gain (g)	Food utilization rate (%)
0 (mg/kg b.w./day)	236.6 ± 29.9	287.5 ± 30.1	49.83 ± 5.07	34.75 ± 3.72
50 (mg/kg b.w./day)	241.7 ± 35.3	281.9 ± 28.9	49.35 ± 5.32	33.98 ± 2.17
100 (mg/kg b.w./day)	238.8 ± 27.4	289.6 ± 30.6	50.02 ± 4.78	35.03 ± 3.08
150 (mg/kg b.w./day)	239.5 ± 26.2	289.2 ± 32.8	49.17 ± 5.19	33.47 ± 2.96
200 (mg/kg b.w./day)	240.5 ± 31.1	288.1 ± 30.7	50.07 ± 4.53	34.14 ± 3.14
250 (mg/kg b.w./day)	237.2 ± 28.3	289.1 ± 32.1	50.22 ± 4.62	35.38 ± 2.26
300 (mg/kg b.w./day)	235.7 ± 26.7	287.4 ± 29.4	49.43 ± 4.73	35.73 ± 2.51

**Table 2:** Effect of LE on serum aspartate aminotransferase (AST), alanine aminotransferase (ALT), alkaline phosphatase (ALP), total protein (TP), albumin (Alb) and globulin (G) levels in different groups.

Group	AST (U/L)	ALT (U/L)	ALP (U/L)	TP (mg/L)	Alb (mg/L)	G (mg/L)
NC	171.82 ± 13.54	22.13 ± 1.75	123.85 ± 11.08	71.54 ± 4.07	39.61 ± 2.08	31.22 ± 2.02
MC (CCl_4_)	401.45 ± 32.07 b	241.62 ± 2.03 b	401.52 ± 32.98 b	51.26 ± 3.62 b	30.11 ± 1.65 b	19.84 ± 1.54 b

CCl_4_ + LE (100 mg/kg b.w.)	289.32 ± 20.19 d	92.57 ± 7.33 d	299.64 ± 29.83 d	60.05 ± 6.83 d	33.07 ± 2.39 c	23.18 ± 1.42 c

CCl_4_ + LE (150 mg/kg b.w.)	297.14 ± 22.14 d	98.41 ± 6.05 d	316.73 ± 27.05 d	64.03 ± 4.42 d	34.15 ± 2.64 d	25.13 ± 1.66 d

CCl_4_ + LE (300 mg/kg b.w.)	193.17 ± 14.71 d	41.09 ± 2.64 d	198.37 ± 12.56 d	69.32 ± 4.91 d	38.53 ± 3.15 d	29.94 ± 2.06 d

CCl_4_ + bifendate	253.05 ± 19.06 d	52.61 ± 3.35 d	194.02 ± 10.47 d	66.38 ± 7.03 d	36.64 ± 1.96 d	30.17 ± 2.36 d

b *P* < 0.01, compared with NC group;

c *P* < 0.01;

d *P* < 0.01, compared with CCl_4_-treated group. NC: normal control, MC: model control.

**Table 3:** Effect of LE on liver index in different groups.

Group	Liver index
NC	3.41 ± 0.28
MC (CCl_4_)	4.95 ± 0.22 b
CCl_4_ + LE (100 mg/kg b.w.)	4.34 ± 0.38 c
CCl_4_ + LE (150 mg/kg b.w.)	4.03 ± 0.42 c
CCl_4_ + LE (300 mg/kg b.w.)	3.56 ± 0.31 d
CCl_4_ + bifendate	3.48 ± 0.29 d

b *P* < 0.01, compared with NC group;

c *P* < 0.05;

d *P* < 0.01, compared with CCl_4_-treated group; NC: normal control.

**Table 4:** Effect of LE on liver hydroxyproline levels in different groups.

Group	Hyp (μg·g^−1^)
NC	99.32 ± 7.05
MC (CCl_4_)	413.17 ± 33.76 b
CCl_4_ + LE (100 mg/kg b.w.)	342.32 ± 20.17 d
CCl_4_ + LE (150 mg/kg b.w.)	306.15 ± 26.08 d
CCl_4_ + LE (300 mg/kg b.w.)	174.63 ± 13.64 d
CCl_4_ + bifendate	149.27 ± 11.07 d

b *P* < 0.01, compared with NC group;

d *P* < 0.01, compared with CCl_4_-treated group; NC: normal control.

**Table 5:** Effect of LE on liver malondialdehyde (MDA) and glutathione (GSH) levels in different groups.

Group	MDA (nmol/mg Prot)	GSH (mg/g Prot)
NC	4.32 ± 2.13	154.29 ± 13.29
MC (CCl_4_)	8.54 ± 4.83 b	88.16 ± 6.72 b
CCl_4_ + LE (100 mg/kg b.w.)	7.39 ± 4.51 c	104.32 ± 10.63 d
CCl_4_ + LE (150 mg/kg b.w.)	6.01 ± 4.21 d	127.39 ± 11.05 d
CCl_4_ + LE (300 mg/kg b.w.)	4.17 ± 3.09 d	150.73 ± 13.29 d
CCl_4_ + bifendate	5.73 ± 3.71 d	144.37 ± 11.73 d

b *P* < 0.01, compared with NC group;

c *P* < 0.05,

d *P* < 0.01, compared with CCl_4_-treated group; NC: normal control.

**Table 6:** Effect of LE on liver antioxidant enzymes activities in different groups.

Group	SOD (U/mg prot)	CAT (U/mg prot)	GSH-Px (U/mg prot)	GR (U/g prot)	GST (nmol/min/mg prot)
NC	164.9 ± 12.67	32.65 ± 3.05	27.51 ± 1.85	31.43 ± 1.65	43.54 ± 4.21
MC (CCl_4_)	83.2 ± 5.09 b	16.93 ± 1.52 b	10.73 ± 1.05 b	13.16 ± 1.25 b	21.34 ± 1.85 b
CCl_4_ + LE (100 mg/kg b.w.)	103.9 ± 8.01 d	20.11 ± 1.67 c	15.75 ± 1.93 c	21.85 ± 1.21 d	31.54 ± 2.86 d
CCl_4_ + LE (150 mg/kg b.w.)	114.5 ± 8.23 d	22.07 ± 1.88 d	19.32 ± 1.43 d	22.43 ± 1.07 d	34.21 ± 2.53 d
CCl_4_ + LE (300 mg/kg b.w.)	159.2 ± 11.72 d	30.57 ± 2.53 d	26.19 ± 1.97 d	30.41 ± 2.63 d	41.83 ± 3.92 d
CCl_4_ + bifendate	142.1 ± 10.61 d	27.58 ± 2.22 d	24.62 ± 2.31 d	29.42 ± 1.84 d	36.1 ± 4.17 d

b *P* < 0.01, compared with NC group;

c *P* < 0.05;

d *P* < 0.01, compared with CCl_4_-treated group; NC: normal control.

**Table 7:** Effect of LE on serum TNF-α levels in different groups.

Group	TNF-α (pg/mL)
NC	202.15 ± 17.84
MC (CCl_4_)	993.14 ± 85.13 b
CCl_4_ + LE (100 mg/kg b.w.)	831.66 ± 75.42 d
CCl_4_ + LE (150 mg/kg b.w.)	703.12 ± 65.09 d
CCl_4_ + LE (300 mg/kg b.w.)	483.19 ± 37.24 d
CCl_4_ + bifendate	503.91 ± 46.18 d

b *P* < 0.01, compared with NC group;

d *P* < 0.01, compared with CCl_4_-treated group; NC: normal control.
